# MiDAS: the field guide to the microbes of activated sludge

**DOI:** 10.1093/database/bav062

**Published:** 2015-06-27

**Authors:** Simon Jon McIlroy, Aaron Marc Saunders, Mads Albertsen, Marta Nierychlo, Bianca McIlroy, Aviaja Anna Hansen, Søren Michael Karst, Jeppe Lund Nielsen, Per Halkjær Nielsen

**Affiliations:** Center for Microbial Communities, Department of Chemistry and Bioscience, Aalborg University, Aalborg East DK-9220, Denmark

## Abstract

The Microbial Database for Activated Sludge (MiDAS) field guide is a freely available online resource linking the identity of abundant and process critical microorganisms in activated sludge wastewater treatment systems to available data related to their functional importance. Phenotypic properties of some of these genera are described, but most are known only from sequence data. The MiDAS taxonomy is a manual curation of the SILVA taxonomy that proposes a name for all genus-level taxa observed to be abundant by large-scale 16 S rRNA gene amplicon sequencing of full-scale activated sludge communities. The taxonomy can be used to classify unknown sequences, and the online MiDAS field guide links the identity to the available information about their morphology, diversity, physiology and distribution. The use of a common taxonomy across the field will provide a solid foundation for the study of microbial ecology of the activated sludge process and related treatment processes. The online MiDAS field guide is a collaborative workspace intended to facilitate a better understanding of the ecology of activated sludge and related treatment processes—knowledge that will be an invaluable resource for the optimal design and operation of these systems.

**Database URL:** http://www.midasfieldguide.org

## Introduction

Activated sludge is a wastewater treatment technology that employs a diverse consortium of microbes for the removal of nutrients from wastewater streams that would otherwise contribute to the eutrophication of the receiving water bodies (1). Increasingly, waste streams are also seen as a valuable resource, e.g. for production of bioenergy or nutrient recovery (2). A deep understanding of the microbial communities and dynamics in treatment systems is a powerful tool for process optimization and design (3, 4).

With the advent of amplicon sequencing of the 16S rRNA gene, the diversity within the microbial communities can now be sampled sufficiently to describe the composition and dynamics of most abundant organisms (5). However, to understand the relationship between the population dynamics and operational parameters of the system, a functional role must be attributed to each organism. Many of the abundant microbes in the activated sludge ecosystem have been investigated with culture-dependent genetic and physiological methods, enrichment in lab-scale reactors, culture-independent molecular approaches or *in situ* physiological studies (reviewed elsewhere (6, 7)). However, many abundant microbes still remain to be studied in greater detail to get a comprehensive and encompassing understanding of the ecosystem.

A putative function can be proposed by classifying 16 S rRNA gene amplicons to a genus or species, which has been characterized, for which the typical function in the ecosystem is known. Classification is usually done by comparison of the unknown sequences to a known reference set with a defined taxonomy. Three public 16 S ribosomal RNA gene sequence databases are routinely used to classify environmental sequences: Greengenes (8), SILVA (9) and RDP (10). Each database is large and the coverage of the known sequence diversity is extensive.

It is important for putative functional assignment that sequences are at least classified to the genus level, because putative functional annotation for higher taxonomic levels is uncertain for all but few phenotypes (11). However, surveys of full-scale systems show that a substantial portion of the sequences are not classified at the genus level, applying the available taxonomic databases (12). Such poor classification can be due to limitations in the phylogenetic information of the query or database sequences; however, in many cases, it is simply due to the name of closely related reference sequences not being fully annotated at all levels in the taxonomy. Genus-level classifications are currently almost exclusively restricted to those with valid published or candidate names, which excludes many environmentally important organisms. The number of genera with valid and candidate names is 2001 and 110, respectively (as of 1 August 2013 (13)). In contrast, based on the predicted bacterial diversity, the total number is estimated to be at least 61 000 (14). Presently, taxonomic annotation is a manual task and improving annotation across the entire microbial database is a substantial undertaking. A provisional solution has been to focus manual annotation on those organisms that are abundant in a particular habitat, i.e. the Human Oral Microbiome Database (HOMD) (15).

The Microbial Database for Activated Sludge (MiDAS) presented here provides a curated taxonomy for abundant and important microorganisms and integrates it into a community knowledge web platform about the microbes in activated sludge. The MiDAS taxonomy proposes putative names for each genus-level-taxon that can be used as a common vocabulary for all researchers in the field. The online MiDAS field guide links the identity of annotated genera to details about their morphology, diversity, physiology and distribution. MiDAS is intended as a collaborative workspace, available for researchers and wastewater treatment practitioners, to facilitate a better understanding of the ecology of this biotechnologically important ecosystem.

## The MiDAS microorganisms

The MiDAS database aims to provide taxonomic assignment and associated physiological information profiles for the abundant and process critical genera in activated sludge treatment systems. The starting list of 152 genera has been populated by organisms found to be abundant and/or important in full-scale treatment plants, based on extensive surveys with fluorescence *in situ* hybridization (FISH) (6, 16) and 16 S rRNA gene amplicon sequencing. Although these surveys have focused on wastewater treatment plants (WWTPs) situated in Denmark, recent work has indicated that the abundant organisms are common to treatment plants globally (Nierychlo,M., Nielsen,P.H. et al., unpublished results).

**Figure 1.**
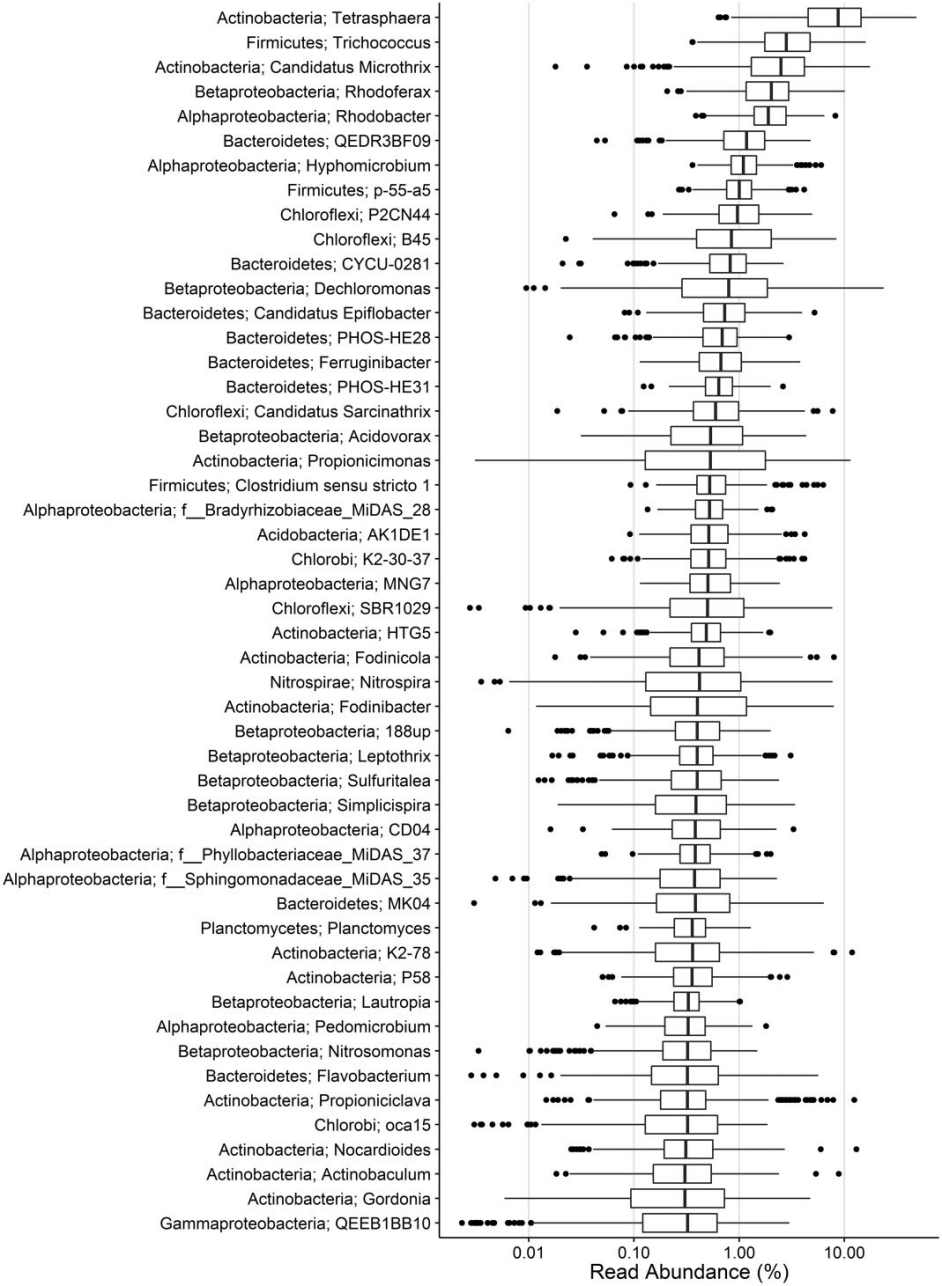
Boxplot showing the abundance of the top 50 genera of the MiDAS amplicon survey. On average, 55% (sd 7%, *n* = 396) of the total sequence reads for each plant classify to these genera. Phylum- and genus-level classification is given in the left. Where genus-level classification is absent, the lowest taxonomic level is given along with the MiDAS reference OTU. See Supplementary information for further details.

We have recently applied 16 S rRNA gene amplicon (V1-3 region) analysis to survey 20 full-scale activated sludge WWTPs in Denmark over a period of 8 years (see Supplementary information for details). The top 50 genera by median abundance are shown in Figure 1**.** The top 100 operational taxonomic units (OTUs) (97% similarity) by median abundance made up on average 50% (sd 7%, *n* = 396) of the total reads in each plant. These OTUs were used to guide the curation of the MiDAS taxonomy. Due to the inherent biases associated with amplicon sequencing (17), more direct measures, such as FISH, will help to evaluate these estimated abundances. Nonetheless, the inclusion of the most frequently observed taxa, by the commonly applied amplicon sequencing method, provides a list of potentially important organisms that can be targeted for further investigation.

Also included in the database are a number of well-studied organisms that were not commonly abundant in the amplicon-based studies, but which have previously been documented as important in some plants. These include, for example: *Skermania,* associated with ‘foaming’ incidents (18); the ‘*Ca.* Accumulimonas*’,* putative polyphosphate accumulating organisms (PAO) previously referred to as ‘*Ca.* Halomonas phosphatis’ (19); and *Brocadia* (20), important ammonia oxidizing bacteria (anammox) involved in nitrogen removal in some new process designs. Additional organisms will be added as other process-relevant organisms are discovered. Although the current version has focused on organisms in full-scale nutrient removal activated sludge systems, it also covers most microorganisms important for related systems, i.e. aerobic granules, membrane bioreactors and influent wastewater communities. Future versions of the database also plan to cover the organisms relevant to the anaerobic digestion component systems often incorporated into wastewater treatment facilities.

## Manual curation of the MiDAS taxonomy

The MiDAS taxonomy is a version of the SILVA database (Release 119, Ref NR 99) that is refined for the classification of activated sludge organisms, rather than being an independent divergent taxonomy. Of the commonly applied taxonomies, SILVA was selected as the base taxonomy as it has a regular release schedule for new sequences, along with an ARB database (21), allowing for manual curation.

The delineation of novel taxa in the MiDAS taxonomy was informed by the recommended similarity cut-off values of Yarza *et al.* (14) for monophyletic groups in the ARB base tree. The choice of sequence clades to name was guided by the position of representative OTU sequences, added to the SILVA (Release 119, Ref NR 99) base tree with the ‘ARB parsimony insertion tool’, and their closest full-length sequence (percentage similarity). Placement of OTUs was improved slightly by the removal of poor quality sequences (color group 1 of the SILVA ARB file) (see Supplementary information), so these were excluded from the MiDAS taxonomy. Most of the clades without annotations at the genus level were known only by their 16 S rRNA gene sequence in the database. These putative phylotypes were given a temporary name derived from the clone identifier of the oldest representative sequence of the clade present in the database. The same approach is used by Greengenes (Hugenholtz,P., personal communication*.*). This naming system provides temporary identifiers for these presumably ecologically important organisms until they receive a candidate or approved name. In general, the classifications of the MiDAS taxonomy are not suggested to be authoritative but seek to provide a common vocabulary for future work aiming to characterize these phylotypes.

Currently, the MiDAS database uses the genus as the functional unit and assumes that genera act as ecotypes with a coherent phenotype. However, there is documented diversity amongst coexisting members of the same genus. Where resolution allows, the MiDAS taxonomy was extended to the ‘species’ taxonomic level to cover defined phenotypically distinct subgroups, such as sublineages of the *Nitrospira* (22). MiDAS OTUs are also available for download to be used as a point of reference – this will provide the possibility of accumulating any pertinent observed OTU-specific information.

### Assessment of the MiDAS taxonomy

To demonstrate the improved annotation for the top 100 OTUs in the MiDAS taxonomy, the same sequences were classified with other available taxonomies (Table 1). Genus-level classification was the highest for the MiDAS taxonomy (91%), followed by RDP (53%), SILVA (49%) and Greengenes (38%). Although RDP was marginally better than SILVA and Greengenes, it did not perform well for higher-level classifications, with 9% of the OTUs not classified to a phylum. Despite manual annotation for these genera in the MiDAS taxonomy, some amplicons could not be confidently assigned to a genus due to limitations in the resolution of 16 S rRNA gene for the V1-3 region (Table 1) (see Yarza *et al.* (14)). This was particularly a problem for some families, such as the *Comamonadaceae* and *Phyllobacteriaceae*. Classification of amplicons will in general likely improve as future advances in sequencing technology allow high-throughput sequencing of longer fragments. Most of the genus-level taxa unique to the MiDAS taxonomy are uncultured phylotypes for which there is no known physiological information. However, there are some important genera, annotated in the MiDAS taxonomy only, which have previously been defined and characterized using *in situ* methods, such as microautoradiography (MAR)-FISH.

**Table 1.** Comparison of taxonomies for the classification of the top-100 MiDAS OTUs

Taxonomy	Classification at phylogenetic level (%)					
	Kingdom	Phylum	Class	Order	Family	Genus
RDP^a^	100	91	87	84	74	53
Greengenes^b^	100	100	100	98	76	38
SILVA^c^	100	100	97	91	87	49
MiDAS^d^	100	100	100	99	99	91

^a^Ribosomal Database Project: Release 11, update 3 (10). ^b^Greengenes: Release May 2013 (8). ^c^SILVA: Release 119, Ref NR 99 (9). ^d^MiDAS: Release 1.20. For further details see Supplementary information.

Application of the MiDAS taxonomy particularly improved classification of members of the phylum *Chloroflexi*. Along with the actinobacterial ‘*Ca.* Microthrix’, members of *Chloroflexi* are the most abundant filamentous organisms associated with sludge settleability problems known as bulking (23). None of the nine most abundant MiDAS-OTUs (of the top 100) that classified to the phylum *Chloroflexi* had genus-level classification with the native SILVA taxonomy. Several of these are associated with Eikelboom morphotypes (24), which have often been associated with bulking and foaming episodes (25) (see Table 2). The abundant genus-level taxa are spread across four classes (*Anaerolineae, Caldilineae, Ardenticatenia* and SJA-15), making it highly unlikely that they respond in the same way to operational conditions. Sludge-derived phylotypes have previously been associated with hydrolysis of organic material (26), denitrification (27), fermentation (28) and nitrite oxidation (29), highlighting the value of genus-level classification and characterization in understanding the dynamics that determine the ecology of members of the phylum. Such important information is currently overlooked when other popular taxonomies are applied.

**Table 2.** Classification of selected Chloroflexi phylotypes with different taxonomies

Eikelboom morphotype^a^	Taxonomy	Classification				
		Phylum	Class	Order	Family	Genus
0092 (30)	RDP^b^	*Chloroflexi*	*Anaerolineae*	*Anaerolineales*	*Anaerolineaceae*	
	Greengenes^c^	*Chloroflexi*	*Anaerolineae*	DRC31		
	SILVA^d^	*Chloroflexi*	*Ardenticatenia*			
	**MiDAS^e^**	***Chloroflexi***	***Ardenticatenia***	**419**	**2-1**	**B45**
0803 (27)	RDP	*Chloroflexi*	*Caldilineae*	*Caldilineales*	*Caldilineaceae*	*Caldilinea*
	Greengenes	*Chloroflexi*	*Anaerolineae*	*Caldilineales*	*Caldilineaceae*	*Caldilinea*
	SILVA	*Chloroflexi*	*Caldilineae*	*Caldilineales*	*Caldilineaceae*	
	**MiDAS**	***Chloroflexi***	***Caldilineae***	***Caldilineales***	***Caldilineaceae***	**P2CN44**
0914 (31)	RDP	*Chloroflexi*	*Caldilineae*	*Caldilineales*	*Caldilineaceae*	
	Greengenes	*Chloroflexi*	*Anaerolinea*	SHA-20		
	SILVA	*Chloroflexi*				
	**MiDAS**	***Chloroflexi***	**SJA-15**	**1-20**	**1-20**	***Ca.* Sarcinathrix**
1851 (28, 32)	RDP	*Chloroflexi*	*Chloroflexia*	*Chloroflexales*	*Chloroflexineae*	*Roseiflexus*
	Greengenes	*Chloroflexi*	*Chloroflexi*	*Roseiflexales*	*Kouleothrixaceae*	*Kouleothrix*
	SILVA	*Chloroflexi*	*Chloroflexia*	*Chloroflexales*	*Roseiflexaceae*	*Roseiflexus*
	**MiDAS**	***Chloroflexi***	***Chloroflexia***	***Chloroflexales***	***Roseiflexaceae***	***Kouleothrix***

^a^Associated morphotype of selected phylotype. ^b^Ribosomal Database Project: Release 11, update 3 (10). ^c^Greengenes: Release May 2013 (8). ^d^SILVA: Release 119, Ref NR 99 (9). ^e^MiDAS: Release 1.20.

In addition, the classification of many of the dominant OTU sequences of the MiDAS survey varied greatly when different reference taxonomies were applied. For example, the four phylotypes shown in Table 2 are classified to different phylogenetic classes depending on the reference taxonomy. Cross-study comparison is difficult—or impossible—when different taxonomies are applied, emphasizing the value in a common environment-specific taxonomy such as MiDAS.

## MiDAS field guide web resource

The MiDAS field guide is available as a web resource (www.midasfieldguide.org). The website provides a searchable database of information about each abundant and/or important genus of activated sludge plants. Information is referenced, with the database acting as a central, online repository for current knowledge about activated sludge organisms.

**Figure 2.**
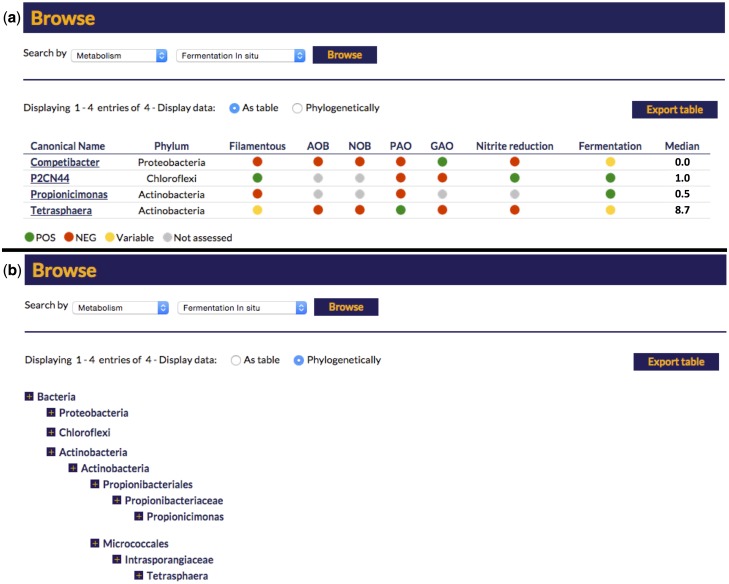
Screen capture of the search options for the online MiDAS field guide. (a) A ‘metabolism’-based search for *in situ* fermentation positive organisms and (b) the same search option with the results presented in a hierarchal phylogeny.

The search function of the website provides a number of entry points to the individual genus descriptions. The genus names can be listed alphabetically or browsed within the phylogenetic hierarchy (see Figure 2). The data fields for each entry are displayed as a table, which can be filtered by each field. Thus, the entries can be sorted by, for example, functional guild or morphology.

### Taxon profiles

Detailed profiles are available for all selected taxa. Descriptions include referenced information on the classification, morphology, metabolism, diversity, targeting FISH probes and distribution of each genus. The details provided for these sections are as follows:

*Genus names:* A taxon’s name status can be either validly published and ‘approved’, according to the *International Code of Nomenclature of Bacteria* (33); ‘candidatus’, for partially characterized organisms (34); or ‘unpublished’, for proposed identifiers given to uncultured phylotypes in the absence of an approved name. Alternate names are included if there are antecedent synonyms or for taxa that are merged in the MiDAS taxonomy.

*16 S rRNA gene copy number*: Putative copy numbers are estimated from available genomes. Such values should be considered in the interpretation of amplicon-sequencing data given that, relative to other taxa present, they influence the determined abundance (35).

*Genomes:* Reference(s) to available genomes are listed here. Genomic information will become increasingly useful as metagenomic population binning methods allow access to uncultured genera (36). Given that there are currently few genomes that represent the dominant genomes *in situ* (37), it is the intention that future versions of the database somehow distinguish relevant genomes.

*Morphology:* Observed ability of the organism to form filaments and microcolonies. Microcolony and filament-forming bacteria make up important structural components of the activated sludge floc that determine the settling properties of the sludge (38, 39). In addition, overgrowth of filamentous bacteria increases interfloc bridging, resulting in poor sludge settleability and bulking (38).

*Metabolism:* Metabolic summaries are given with classification of the organisms into important functional guilds, including the ammonia and nitrite oxidizing bacteria (AOB and NOB), anaerobic ammonium oxidizers (Anammox), polyphosphate and glycogen-accumulating organisms (PAO and GAO), denitrifiers (nitrite reduction), fermenters and other anaerobic activities (i.e. iron or sulphate reduction). A more general description of the phenotypic properties of the genus is also given. *In situ* evidence for traits is separated from pure culture and genomic information (collectively represented by the field ‘other evidence’); given the latter two represent only the potential of the organism for a phenotypic trait *in situ*. Annotated pathways are not always expressed *in situ* (40), and activated sludge organisms generally appear to be much more specialized *in situ* than when grown in pure culture (41). Furthermore, isolates and genomes may not represent the abundant environmental species of the genus (37)*.*

*Distribution:* Correlations for operational conditions, process design and geographic location are listed. The median read abundance and the 10 and 90 % quantiles, based on the MiDAS amplicon survey, are also given for each genus. Reference OTU identifiers from the MiDAS survey are also provided, including the top 100 and all present at >0.1% in at least one plant.

*Diversity:* Known diversity within the genus, such as clades/subgroups and, distinct MiDAS OTUs and species, are given here. For some genera, subgroups with varied phenotypic properties are reported i.e. the varied optimal nitrite concentrations for the sublineage of the NOB *Nitrospira* (22).

*FISH probes:* Suggested FISH probes for *in situ* analyses of the genera. Further *in situ* characterization is facilitated by the application of these probes, which for the uncultured phylotypes is the only source of phenotypic information.

### Other resources and licensing information

The QIIME-formatted (42) MiDAS taxonomy file and the reference OTU sequences, used to curate the taxonomy, are available for download from the web platform. Also available are several relevant protocols, i.e. DNA extraction from sludge and sample preparation for amplicon sequencing. Available protocols have been extensively validated for sludge samples (17) and are routinely updated. Each genus entry has an integrated comment function, hosted by Disqus (https://disqus.com/), and users in the field are encouraged to draw attention to new data—potentially highlighting their own work—which can be incorporated into the database.

The MiDAS website is freely accessible and is licensed under the Creative Commons Attribution-ShareAlike 4.0 International license. Given the MiDAS taxonomy is a modified version of the SILVA database, users should comply with the terms of use outlined by SILVA (http://www.arb-silva.de/silva-license-information/).

## Concluding remarks

The MiDAS field guide is intended as a collaborative platform for researchers, consultants and wastewater treatment practitioners, to improve the classification of unknown organisms and link these names to the wealth of present and future functional information about their ecology in activated sludge and related systems. The MiDAS genus names proposed can provide a common vocabulary for all researchers in the field, facilitating the exchange of data and benefit studies into the ecology of these industrially important ecosystems. MiDAS is an ongoing project that will be periodically updated to reflect advances in the field.

## Supplementary Data

Supplementary data are available at *Database* Online.

## Funding

This study was supported by Danish Wastewater Association, Krüger A/S, Kemira A/S and ∼50 municipal wastewater treatment plants (the ‘Microbial Database’), Aalborg University and the Innovation Fund Denmark (EcoDesign-MBR) [grant number 09-067230]. Funding for open access charge: 09-067230.

*Conflict of interest*. None declared.

## Supplementary Material

Supplementary Data
