# Supplementary material for: MiDAS: the field guide to the microbes of activated sludge
Source: Database (Oxford). 2015 Jun 27;2015:bav062. doi: 10.1093/database/bav062 (PMC4483311; doi:10.1093/database/bav062)
Supplement: Supplementary Data [file supp_2015_bav062_index.html]

MiDAS: the field guide to the microbes of activated sludge — Supplementary Data 

# MiDAS: the field guide to the microbes of activated sludge

## Supplementary Data

files

- Supplementary Data - pdf file
