# Supplementary material for: MiDAS: the field guide to the microbes of activated sludge
Source: Database (Oxford). 2015 Jun 27;2015:bav062. doi: 10.1093/database/bav062 (PMC4483311; doi:10.1093/database/bav062)
Supplement: Supplementary Data [file supp_bav062_MiDAS_Suppl_Info_Online.pdf]

Supplementary information

MiDAS: the Field Guide to the Microbes of Activated Sludge

Simon Jon McIlroy, Aaron Marc Saunders, Mads Albertsen, Marta Nierychlo, Bianca McIlroy, Aviaja A. Hansen, Søren Michael Karst, Jeppe Lund Nielsen and Per Halkjær Nielsen

The MiDAS amplicon survey of Danish wastewater treatment plants (Albertsen, M., Nielsen, P.H. and others, in prep.)

Activated sludge was sampled from the aeration tanks of 55 different Danish wastewater treatment plants. Sampling was carried out up to four times a year from 2006 to 2013 resulting in a total of 574 samples.

DNA extraction was conducted using the FastDNA spin kit for soil (MP biomedical) according to the manufactures instructions, except the bead beating was increased to 4x40s at 6 m/s using a FastPrep FP120 (MP Biomedicals). The procedure for bacterial 16S rRNA amplicon sequencing targeting the V1-3 variable region was modified from Caporaso and colleagues (1). Briefly, 10 ng of extracted DNA was used as template and the PCR reaction (25 µL) contained dNTPs (400nM of each), $MgSO_4$ (1.5 mM), Platinum® Taq DNA polymerase HF (2mU), 1X Platinum® High Fidelity buffer (Thermo Fisher Scientific,) and a pair of barcoded library adaptors (400 nM). V1-3 primers: 27F AGAGTTTGATCCTGGCTCAG and 534R ATTACCGCGGCTGCTGG (2). Thermo cycler settings: Initial denaturation at 95 °C for 2 min, 30 cycles of 95°C for 20s, 56°C for 30s, 72°C for 60s and final elongation at 72°C for 5 min. All PCR reactions were run in duplicate and pooled afterwards. The amplicon libraries were purified using the Agencourt® AMpure XP bead protocol (Beckmann Coulter, Brea, CA, USA) with the following exceptions: the sample/bead solution ratio was 5/4 and the purified DNA was eluted in 33 µL nuclease-free water. Library concentration was measured with Quant-iT™ HS DNA Assay (Thermo Fisher Scientific) and quality validated with a Tapestation 2200 using D1K ScreenTapes (Agilent). Based on library concentrations and calculated amplicon sizes the samples were pooled in equimolar concentrations and diluted to 4 nM. The library pool was sequenced on a MiSeq (Illumina) using a MiSeq Reagent kit v3 (2x300 PE) following the procedure in (3), with exception of 10% Phix control library (Illumina) spike-in and final library loading concentration of 20 pM. A complete detailed DNA extraction and amplicon sequencing protocol can be obtained from www.midasfieldguide.org/en/protocols.

16S rRNA data processing

All sequenced sample libraries were subsampled to 50 000 raw reads and low quality reads removed using Trimmomatic v. 0.32 with the settings SLIDINGWINDOW:1:3 and MINLEN:275 (4).

Forward and reverse reads were merged using FLASH v. 1.2.7 (5), with the settings -m 25 -M 200 and afterwards merged reads smaller than 425 bp or larger than 525 bp were discarded.

All merged reads were screened for phiX contamination using usearch7.0.1090 (6), with standard settings and all matching reads removed. The potential phiX contamination is due to the use of an un-indexed phiX as a quality control, which can result in index-carryover from nearby clusters with indexes.

The merged reads were de-replicated and formatted for use in the uparse workflow (7). The merged reads were clustered using the usearch v. 7.0.1090 –cluster_otus with default settings. OTU abundance was estimated using the usearch v. 7.0.1090 –usearch_global with –id 0.97. Taxonomy was assigned using the RDP classifier (8) as implemented in the parallel_assign_taxonomy_rdp.py script in QIIME (1) using MiDAS taxonomy version 1.20 which is based on the SILVA, Release 1.19 taxonomy (9).

Comparison with other taxonomies
The Greengenes taxonomy v. 13_8 at 97% OTU level (10) was used with the parallel_assign_taxonomy_rdp.py script in QIIME (1). The RDP taxonomy assigned using the RDP database version 11 update 3 with the RDP classifier (11). The ARB version of SILVA, Release 119, Ref NR 99 (9), was downloaded and the 534968 sequences and taxonomy strings exported in .fasta format. In addition, in order to assess the impact of poor quality sequences on taxonomic assignment, the 62603 sequences with low pintail and/alignment scores (classified as 'color group 1' in the SILVA ARB file) were removed.

Classification of the top 100 OTUs applying the native SILVA taxonomy gave a slightly higher proportion of genus classifications (3%) with the exclusion of poor quality sequences. Many of these suspect sequences are likely chimeric in nature. As such sequences are made up of smaller heterogenous fragments of the 16S rRNA gene (12), they are particularly problematic for the classification of the short amplicons, given the latter will likely not span the chimeric break point and may be highly similar to one of the contributing sequence fragments of the chimera. Sequences of 'color group 1' were therefore excluded from the MiDAS taxonomy.

16S rRNA data analysis and visualization
All data analysis and visualizations were conducted using R (13) through the Rstudio IDE (http://www.rstudio.com/). OTU counts and associated taxonomic assignments were imported and merged to a phyloseq object (14) and analysed using the ampvis R package (https://github.com/MadsAlbertsen/ampvis). The median abundance of the

OTUs was determined from the 20 WWTPs with most samples in the database (n=396).

Data availability
The raw V13 16S rRNA amplicon sequences are part of the MiDAS dataset (www.midasfieldguide.org) available in European Nucleotide Archive under study PRJEB8105. All processed data is included in the ampvis R package as a data object: data (MiDAS_1.20).

References

1. Caporaso, J. G., Kuczynski, J., Stombaugh, J., et al. (2010) QIIME allows analysis of high-throughput community sequencing data. *Nat. Methods*, **7**, 335–6.

2. Chen, T., Yu, W.-H., Izard, J., et al. (2010) The Human Oral Microbiome Database: a web accessible resource for investigating oral microbe taxonomic and genomic information. *Database (Oxford).*, **2010**, baq013.

3. Caporaso, J. G., Lauber, C. L., Walters, W. A., et al. (2012) Ultra-high-throughput microbial community analysis on the Illumina HiSeq and MiSeq platforms. *ISME J.*, **6**, 1621–4.

4. Lohse, M., Bolger, A. M., Nagel, A., et al. (2012) RobiNA: a user-friendly, integrated software solution for RNA-Seq-based transcriptomics. *Nucleic Acids Res.*, **40**, W622–7.

5. Magoč, T. and Salzberg, S. L. (2011) FLASH: fast length adjustment of short reads to improve genome assemblies. *Bioinformatics*, **27**, 2957–63.

6. Edgar, R. C. (2010) Search and clustering orders of magnitude faster than BLAST. *Bioinformatics*, **26**, 2460–1.

7. Edgar, R. C. (2013) UPARSE: highly accurate OTU sequences from microbial amplicon reads. *Nat. Methods*, **10**, 996–8.

8. Wang, Q., Garrity, G. M., Tiedje, J. M., et al. (2007) Naive Bayesian classifier for rapid assignment of rRNA sequences into the new bacterial taxonomy. *Appl. Environ. Microbiol.*, **73**, 5261–7.

9. Quast, C., Pruesse, E., Yilmaz, P., et al. (2013) The SILVA ribosomal RNA gene database project: improved data processing and web-based tools. *Nucleic Acids Res.*, **41**, D590–6.

10. McDonald, D., Price, M. N., Goodrich, J., et al. (2012) An improved Greengenes taxonomy with explicit ranks for ecological and evolutionary analyses of bacteria and archaea. *ISME J.*, **6**, 610–8.

11. Cole, J. R., Wang, Q., Fish, J. A., et al. (2014) Ribosomal Database Project: data and tools for high throughput rRNA analysis. *Nucleic Acids Res.*, **42**, D633–42.

12. Liesack, W., Weyland, H. and Stackebrandt, E. (1991) Potential risks of gene amplification by PCR as determined by 16S rDNA analysis of a mixed-culture of strict barophilic bacteria. *Microb. Ecol.*, **21**, 191–8.

13. R Core Team (2014) R: A language and environment for statistical computing. *R Foundation for Statistical Computing, Vienna, Austria.* (http://www.R-project.org/).

14. McMurdie, P. J. and Holmes, S. (2013) phyloseq: an R package for reproducible interactive analysis and graphics of microbiome census data. *PLoS One*, **8**, e61217.
